# Bacterial and fungal co-colonization alters *Malassezia restricta* metabolism and host proteolytic activity in reconstructed human epidermis

**DOI:** 10.3389/fmicb.2026.1927213

**Published:** 2026-08-24

**Authors:** Eun Sun Lyou, Sang-Hyeon Yoo, Ki-Jong Rhee, David R. Johnson, Tae Kwon Lee

**Affiliations:** 1 Department of Environmental and Energy Engineering, Yonsei University, Wonju, Republic of Korea; 2 Department of Biomedical Laboratory Science, Yonsei University, Wonju, Republic of Korea; 3 Department of Environmental Microbiology, Swiss Federal Institute of Aquatic Science and Technology (Eawag), Dübendorf, Switzerland; 4 Institute of Ecology and Evolution, University of Bern, Bern, Switzerland

**Keywords:** epidermal barrier, fungal metabolic reprogramming, interkingdom interaction, polymicrobial interaction, trypsin-like activity

## Abstract

**Introduction:**

On the skin, bacteria and fungi form mixed communities whose interactions can reshape both microbial physiology and host tissue, yet the microbial mechanisms remain poorly defined. Although polymicrobial colonization severity often exceeds the effects of individual pathogens in these conditions, the mechanism driving this synergy is unclear.

**Methods:**

We used a three-dimensional reconstructed human epidermis to characterize how co-colonization by *Staphylococcus aureus* and *Malassezia restricta* alters barrier structure.

**Results:**

Co-colonized tissue thickened in the viable layers rather than the stratum corneum, a pattern absent from both monocultures whereas *M. restricta* alone thickened the stratum corneum. Filaggrin redistributed toward the stratum corneum, and cytokine release and loss of cell viability tracked with the presence of *S. aureus*. *M. restricta* shifted its transcriptional program toward reduced oxidative phosphorylation and elevated stress response, yielding 453 differentially expressed genes. Screening these genes for secreted hydrolases returned no candidate that would account for host protease activity. Trypsin-like activity nonetheless rose more than tenfold under co-colonization while remaining at baseline under either species alone.

**Discussion:**

Barrier disruption in this model therefore emerges from the two-species condition rather than from either organism which offers one explanation for why polymicrobial colonization tracks with severity in inflammatory skin disease.

## 1 Introduction

The human skin microbiome includes complex microbial communities, wherein bacterial and fungal species engage in dynamic metabolic and competitive interactions that fundamentally influence cutaneous homeostasis (Byrd et al., 2018; Han and Kim, 2024). While commensal microorganisms typically maintain barrier integrity through colonization resistance and immunomodulatory signaling, dysbiotic shifts toward pathobiont dominance can cause inflammatory dermatoses including atopic dermatitis, seborrheic dermatitis, and psoriasis (Boxberger et al., 2021; Fyhrquist et al., 2019; Park M. et al., 2021; Zheng et al., 2020). Accordingly, recent clinical investigations have established strong correlations between polymicrobial colonization patterns and disease severity (Brown et al., 2022; Lee et al., 2025); however, the molecular mechanisms governing interkingdom microbial interactions and their collective impact on epidermal barrier function remain poorly characterized. This knowledge gap limits understanding of how interkingdom interactions reshape microbial physiology and destabilize the epidermal barrier.

Microbial colonization triggers complex host proteolytic cascades that alter epidermal homeostasis. For instance, *Staphylococcus aureus* colonization activates the kallikrein-kinin system via phenol-soluble modulin-mediated membrane perturbation, initiating serine protease cascades that degrade corneodesmosomes and compromise barrier integrity (Paharik and Horswill, 2016; Williams et al., 2019). Concurrently, *S. aureus*-secreted proteases, including the V8 protease and exfoliative toxins, directly cleave desmoglein-1 and filaggrin and thereby increase endogenous proteolytic activity (Rasquel-Oliveira et al., 2025). Additionally, *Malassezia* fungal species contribute to these distinct pathogenic mechanisms by secreting lipases and proteases that modify stratum corneum (SC) lipid composition and activate pattern recognition receptors, triggering NLRP3 (NOD-like receptor protein 3) inflammasome-mediated interleukin-1β (IL-1β) release. Thus, *Malassezia* colonization disrupts terminal differentiation, impairs filaggrin processing, and induces hyperkeratosis in three-dimensional skin models (Park H. R. et al., 2021). These primary effects are associated with excessive interleukin-6 (IL-6) production, which establishes inflammatory cascades that progressively deteriorate barrier integrity (Sparber and LeibundGut-Landmann, 2017; Sparber et al., 2019). However, studies examining individual pathogen-host interactions failed to explain the clinical observations wherein co-colonization severity exceeds the additive effects. Additionally, whether interkingdom interactions influence host protease activation remains unexplored despite clinical correlations between polymicrobial burden and disease progression.

Moreover, recent co-colonization studies have revealed distinct research trajectories between different polymicrobial systems, highlighting critical mechanistic gaps in barrier-specific pathogenic processes. For instance, *S. aureus*-*Candida* interactions drive synergistic virulence through modulated biofilm architecture and quorum sensing pathways in polymicrobial infections (Paul et al., 2024). In contrast, *Staphylococcus*-*Malassezia* co-colonization studies have primarily focused on transcriptional profiling of physiological adaptations and virulence factor upregulation, identifying the enhanced expression of inflammatory mediators and metabolic pathway alterations without establishing direct mechanistic links to barrier disruption (Lyou et al., 2023; Yang et al., 2023). This disparity between the extensive pathogenic characterization of bacterial and fungal biofilm systems and the predominantly descriptive transcriptional analyses of skin-associated polymicrobial communities highlights a lack of systematic investigations on host tissue responses. Furthermore, current co-culture systems utilizing monolayer keratinocyte models fail to recapitulate the spatial architecture necessary to detect protease gradient formation and layer-specific barrier deterioration. Consequently, it remains unclear whether transcriptional reprogramming during interkingdom interactions is accompanied by host proteolytic activity that exceeds the contribution of either organism alone. In this regard, the three-dimensional reconstructed human epidermis (RHE) offers unique advantages for dissecting polymicrobial pathogenesis, including the preservation of native protease inhibitor gradients, maintenance of differentiation-dependent barrier proteins, and accessibility for layer-specific enzymatic activity quantification. These organotypic systems enable controlled polymicrobial colonization experiments, while maintaining the structural complexity required to detect co-colonization-specific changes within stratified tissue architectures.

Here, we characterize fungal-bacterial co-colonization in 3D-RHE and identify host responses associated with co-colonization. Transcriptomic profiling showed that co-colonization was associated with downregulation of fungal amino acid biosynthesis genes and increased expression of several hydrolase-annotated genes, although no predicted secreted hydrolase was upregulated. Using layer-specific molecular quantification, transcriptomic profiling, and enzymatic assays, we show that interkingdom interaction generates proteolytic activity exceeding the contributions of either species alone. These findings indicate that fungal metabolic adaptation, rather than either organism in isolation, reshapes the proteolytic environment of the epidermis.

## 2 Materials and methods

### 2.1 Culture conditions and inoculum preparation

*Staphylococcus aureus* USA300 JE2 (NR-46543) was provided by the Network on Antimicrobial Resistance in *Staphylococcus aureus* (NARSA) for distribution through the BEI Resources (NIAID, NIH) (Kennedy et al., 2008). The plasmid pCM29, which confers chloramphenicol resistance and constitutively expresses green fluorescent protein (GFP), was obtained from Dr. Alexander Horswill (Pang et al., 2010). This plasmid was introduced into the JE2 cells to generate a GFP-expressing strain. *M. restricta* KCTC 27527 was obtained from the Korean Collection for Type Cultures (KCTC).

*Staphylococcus aureus* was cultured in tryptic soy broth (TSB) supplemented with 25 μg/mL chloramphenicol and incubated overnight (16–20 h) at 37°C with reciprocal shaking at 130 rpm. The bacterial cells were washed twice to remove the residual medium, resuspended in fresh medium, and cultured for another 3 h in a shaking incubator till they achieved exponential growth. *M. restricta* was cultured on Leeming and Notman agar (LNA; pH6, 0.5% glucose, 1% polypeptone, 0.01% yeast extract, 0.8% bile salt, 0.1% glycerol, 0.05% glycerol monotrearate, 0.05% Tween 60, 1.2% agar, and 0.5% sterile whole fat cow milk) at 34°C for 3 days. Fungal cells were freshly harvested from 3-day-old cultures for each experiment. The *S. aureus* and *M. restricta* cells were washed with phosphate buffered saline and resuspended in modified Dixon’s medium (mDixon; pH6.5, 3.6% malt extract, 2% bile salt, 1% Tween 40, 0.6% polypeptone, 0.2% oleic acid, and 0.2% glycerol) and sterilized by autoclaving (Gueho et al., 1996). The cell density of each microbial suspension was adjusted to achieve the target concentration based on OD600 measurements, which was retrospectively confirmed by plating serial dilutions for colony-forming unit (CFU) enumeration. These suspensions were then used to inoculate the RHE.

### 2.2 3 D reconstructed human epidermis culture model

The 3D RHE (KeraSkinTM, Biosolution Co., Seoul, Republic of Korea) was used as a physiologically relevant *in vitro* analog of human epidermal tissues. KeraSkin consists of stratified human keratinocytes that form the SC, SG, SS, and SB layers. These tissues are generated by seeding primary keratinocytes onto 12-mm (0.6 cm^2^) Millicell^®^ inserts (Millipore, MA, USA), which are supplied in sets of 24 inserts on agarose gels. All batches were quality-assured by the manufacturer according to viability (OD_570_ of the negative control within 0.7–1.6, positive control viability ≤ 40%), ET-50, IC-50, and histopathological morphology.

Upon receipt of the KeraSkin™ model (Biosolution Co., Seoul, Republic of Korea), all supplied components (epidermal tissue inserts and 50 mL of culture medium) were verified. The assay medium was pre-warmed in a water bath at 37 °C for 30 min prior to use. During this period, the inserts were visually inspected to confirm tissue integrity, including surface uniformity, absence of excess surface moisture, and absence of air bubbles beneath the membrane. Prior to incubation, 0.9 mL of the pre-equilibrated and pre-warmed KeraSkin™ culture medium was dispensed into each well of sterile 6-well plates, and the tissue inserts were carefully transferred into the wells. Any air bubbles trapped beneath the inserts were removed, and the cultures were incubated in a humidified atmosphere (37 °C, 5% CO_2_) for 22 ± 2 h prior to colonization experiments. No changes in the medium were observed during equilibration.

### 2.3 Microbial colonization methodology using spatial confinement approach

After a 24 h equilibration period, the RHE was aseptically transferred to fresh culture medium and subjected to controlled microbial colonization using a standardized spatial confinement protocol. Briefly, sterile borosilicate glass cloning cylinders (outer diameter 7.7 mm, Cat#90070, SPL, Pocheon, Republic of Korea) were used to create defined colonization zones while preventing lateral microbial migration across the tissue surface. The cylinders were positioned centrally on each RHE insert using sterile forceps maintaining a space of approximately 0.5 mm from the culture well periphery to ensure proper medium circulation and gas exchange. *S. aureus* and *M. restricta* suspensions were diluted and mixed to yield final concentrations of 4 × 10^8^ CFU/ml for *S. aureus* and 1.6 × 10^7^ CFU/ml for *M. restricta*. Subsequently, 30 μL of each microbial preparation was applied within the confined cylinder area, resulting in final inoculation densities of 10^7^ CFU/well for *S. aureus* and 5 × 10^6^ CFU/well for *M. restricta*. These inoculation amounts have previously been used to induce skin inflammation in mice (Kline et al., 2024; Sparber et al., 2019). The experimental design included four distinct conditions: (i) monoculture *S. aureus* colonization (SA), (ii) monoculture *M. restricta* colonization (MR), (iii) co-culture with pre-mixed bacterial-fungal suspensions applied simultaneously (MIX), and (iv) sterile medium controls containing modified Dixon medium alone (CTL). All cultures were maintained at 37 °C under 5% CO_2_ atmosphere for 6 h until visible surface desiccation occurred, at which point the glass cylinders were aseptically removed to allow unrestricted microbial-tissue interactions. The colonized RHE were subsequently incubated for 48 h under standard culture conditions to establish mature microbial biofilms and assess host-microbe interactions. This temporal framework was selected based on preliminary studies that demonstrated optimal biofilm formation and measurable transcriptional responses within this timeframe. The experiment was repeated thrice.

### 2.4 Multi-parameter endpoint analysis

After colonization for 48 h, tissues were systematically harvested from the RHE using sterile 8-mm biopsy punches (SPICA, Republic of Korea) to capture the complete microbial colonization zone. Each tissue section was collected using a hierarchical sampling strategy. A central 4 mm core was fixed in 4% paraformaldehyde (PFA) for 4 h for histological processing and immunohistochemical analysis, while the remaining tissue was bisected for parallel analyses. One segment was placed in 1 mL DEPC-treated water for total RNA extraction for microbial transcriptomic profiling. The second segment was suspended in 500 μL trypsin assay buffer for quantitative proteolytic enzyme activity assessment. This integrated approach enabled the simultaneous evaluation of (i) tissue morphology and protein expression, (ii) cellular metabolic activity, and (iii) microbial gene expression profiles from individual experimental units, eliminating inter-replicate variability while maximizing the analytical output for characterization of host-microbe interactions.

### 2.5 Histopathological analysis and immunofluorescent characterization

Tissue morphology and barrier protein expression were assessed using standardized histological and immunofluorescence protocols. Briefly, the RHEs were fixed in 4% PFA to preserve tissue morphology and protein architecture. Subsequently, the tissues were trimmed to a thickness of approximately 2–3 mm to expose the epithelial surface and ensure uniform paraffin infiltration. The trimmed specimens were placed in cassettes and underwent automated tissue processing for 13 h, including graded ethanol dehydration, xylene clearing, and paraffin infiltration to replace intercellular water with molten paraffin and provide structural rigidity. Thereafter, the resulting blocks were sectioned at a thickness of 4 μm using a microtome, mounted on positively charged glass slides, and dried overnight at 60 °C prior to staining.

The paraffin-embedded sections were deparaffinized, rehydrated, and sequentially stained with hematoxylin and eosin (Sigma-Aldrich, MO, USA). After dehydration and clearing, the slides were mounted using Dako Mounting Medium (Dako, Baar, Switzerland) and imaged under bright-field illumination using a Zeiss Axio Scan.Z1 microscope equipped with a Plan-Apochromat 20×/0.8 M27 objective and a Hitachi HV-F203SCL camera (Carl Zeiss, Jena, Germany).

Sections were prepared for immunofluorescence staining, by antigen retrieval in Tris-EDTA buffer (pH 9.0), followed by blocking with 4% BSA for 30 min. The sections were the incubated with a primary anti-filaggrin antibody (1:400; Abcam, ab81468) for 1 h at room temperature, followed by incubation with an Alexa Fluor 594–conjugated secondary antibody (1:200; Abcam, ab150080) for 30 min. Nuclei were counterstained with DAPI-containing mounting medium (Vector Laboratories, CA, USA). Fluorescence images (DAPI, AF594, and EGFP channels) were acquired using a Zeiss Axio Scan.Z1 microscope fitted with an ORCA Flash camera (Hamamatsu, Japan) under standardized LED illumination at 20× magnification. Morphometric and fluorescence intensity quantifications were performed for ten randomly selected fields per tissue section. Automated image acquisition with standardized exposure settings was used to ensure reproducible measurements across all experimental conditions.

For layer-resolved analysis, filaggrin RawIntDen was quantified separately in the SC, SG, and SS + SB and normalized to layer area. The SC fraction of the total filaggrin signal was calculated as SC RawIntDen/[SC RawIntDen + SG RawIntDen + (SS + SB) RawIntDen].

Histological alterations were assessed by semi-quantitative scoring of coded H&E images. Slides were de-identified and randomized by an independent colleague, and all evaluable fields were relabeled with condition-independent identifiers before assessment. A single investigator blinded to condition scored six to nine randomly selected fields per condition, drawn from two independent RHE inserts. Three features were graded from 0 to 3, namely SG–SS disorganization, SS intercellular clefting, and basal-layer abnormality. SG–SS disorganization and SS intercellular clefting were graded by the extent and distribution of the alteration. Basal-layer abnormality was graded by the regularity and continuity of the basal cell layer along the tissue-support membrane interface. Processing-related spaces, section folds, edge artifacts, and separations within or above the stratum corneum were excluded from the assessment of intercellular clefting. Scoring criteria are given in Supplementary Table 1 and field-level scores in Supplementary Table 2.

### 2.6 Cell viability assessment using MTT-based metabolic activity

The cellular metabolic activity and viability of the colonized RHE cells were assessed using a standardized 3-(4,5-dimethylthiazol-2-yl)-2,5-diphenyltetrazolium bromide (MTT) reduction assay. Briefly, microbial biomass and residual culture medium were removed by ten sequential washes with sterile Dulbecco’s phosphate-buffered saline (DPBS, pH 7.4) to eliminate potential interference from metabolically active microorganisms. The MTT reagent was freshly prepared at 0.4 mg/mL in phenol-red-free Dulbecco’s Modified Eagle Medium (DMEM) and applied to the washed tissue constructs. The reduction reaction proceeded for 3 h at 37 °C under 5% CO_2_ atmosphere in darkened conditions to prevent photodegradation of the tetrazolium compound. Subsequently, the unreacted MTT solution was aspirated and the resulting insoluble purple formazan crystals were solubilized in isopropanol with gentle agitation for 15 min at room temperature. Finally, spectrophotometric quantification was performed at 570 nm using a NanoDrop spectrophotometer (Thermo Fisher Scientific).

### 2.7 Quantitative cytokine profiling using enzyme-linked immunosorbent assay

Pro-inflammatory cytokine secretion profiles were quantified from conditioned culture media collected 48 h post-colonization using an enzyme-linked immunosorbent assay (ELISA) kit (R&D Systems, Minnesota, USA). Specifically, human IL-1β, IL-6, and TNF-α levels were measured according to the manufacturer’s protocols. Highly bound 96-well microplates were coated with capture antibodies specific for each target cytokine overnight at room temperature, followed by blocking with a proprietary reagent diluent to minimize nonspecific binding interactions. Subsequently, 100 μL of the culture supernatants and recombinant cytokine standards were applied to the plates in triplicate and incubated for 2 h at room temperature. After washing, biotinylated detection antibodies were added and incubated for 2 h at room temperature, followed by the application of streptavidin–horseradish peroxidase (HRP) conjugate for 20 min according to the manufacturer’s instructions. The substrate solution was then added and incubated for 20 min at room temperature in the dark to achieve colorimetric development. The reaction was terminated with a stop solution, and the optical density was immediately measured at 450 nm using a Tecan Infinite 200 Pro microplate reader (Tecan Group Ltd., Switzerland) controlled by the i-control software (v1.9.17.0). Cytokine concentrations were determined from standard curves generated by four-parameter logistic regression using the GraphPad Prism software to ensure accurate interpolation across the dynamic range of the assay.

### 2.8 Quantitative proteolytic enzyme activity assessment

Proteolytic enzyme activity within the colonized RHE was systematically quantified using trypsin activity assays as an indicator of microbial pathogenicity and tissue degradation. Briefly, tissue samples suspended in 500 μL of trypsin assay buffer (Biovision Inc., CA, USA) and centrifuged at 13,000 rpm for 10 min at 4 °C to obtain clarified supernatants containing soluble proteolytic enzymes. The chromogenic assay with synthetic peptide substrates specific for trypsin using the Trypsin Activity Colorimetric Assay Kit (Biovision Inc., CA, USA), was then used according to the manufacturer’s instructions. The reaction mixture consisted of trypsin substrate and the assay buffer provided by the kit. Supernatants were transferred to 96-well microplates and incubated with the reaction mixture at 25 °C for 2 h in a light-protected environment to prevent photodegradation. Trypsin cleaved the substrate to generate p-nitroaniline (p-NA), which was detected at 405 nm using a microplate reader. The enzymatic activity was calculated using standard curves constructed from known concentrations of p-NA. The standard absorbance values were fitted to a linear regression model, and p-NA production in each sample was interpolated from the corresponding regression equation. Trypsin activity (mU/mL) was then determined as the change in p-NA concentration over 2 h, normalized to the incubation time and pretreated sample volume (0.05 mL).

### 2.9 Transcriptomic profiling of *M. restricta*

Transcriptome analysis of *M. restricta* was performed on the MR and MIX groups, using two independent RHE inserts per group. The CTL and SA groups were not sequenced, because they contain no *M. restricta*. MR denotes fungal mono-colonization and MIX denotes co-colonization with *S. aureus*, and all comparisons are MIX relative to MR. Total RNA was extracted from the colonized tissue samples using a hybrid extraction protocol using the TRIzol reagent (Invitrogen, MA, USA) for initial cell lysis and lipid removal, followed by purification using RNeasy Mini Kit columns (Qiagen, Maryland, USA). Quality of the extracted RNA was assessed using an Agilent 2100 Bioanalyzer system (Agilent Technologies, Santa Clara, CA, USA) with specific evaluation of RNA integrity number (RIN) values (>7.0), 28S/18S ribosomal RNA ratios, and total RNA concentration to ensure sample suitability for high-throughput sequencing. Strand-specific RNA-seq libraries were prepared using a TruSeq Stranded Total RNA Kit with Ribo-Zero technology (Illumina, San Diego, CA, USA), which was specifically optimized for microbial applications. Library quantification employed a quantitative polymerase chain reaction (PCR) methodology following standardized Illumina protocols, with individual libraries normalized to achieve equimolar pooling for multiplexed sequencing. High-throughput sequencing was performed using the NovaSeq 6000 platform (Illumina, CA, USA) at Macrogen, Inc., (Seoul, Republic of Korea). No *M. restricta*-specific enrichment or normalization was performed before library preparation, so fungal transcripts were retained as part of the rRNA-depleted total RNA libraries.

### 2.10 Computational transcriptome analysis and functional annotation pipeline

The reads were quality filtered and adapter-trimmed before alignment to the *M. restricta* reference genome (GCF_003290485.1_ASM329048v1) using HISAT2 (version 2.1.0) (Langmead et al., 2009). Fungal reads accounted for 5.21% and 5.62% of the two MR libraries and 10.47% and 8.16% of the two MIX libraries. Gene expression was quantified using StringTie (version 2.1.3b) with a known gene annotation. Of the 4,544 annotated genes, 150 with a zero count in at least one sample were excluded, leaving 4,394 genes for analysis. DEG analysis was performed using the EdgeR statistical framework with negative binomial models to identify genes that were significantly altered between the axenic and co-colonized conditions. Differentially expressed genes were defined as an absolute fold change of at least 2 and a raw *p*-value below 0.05. Functional annotation and pathway enrichment analyses were performed using the KEGG database integrated with the Database for Annotation, Visualization, and Integrated Discovery (DAVID) bioinformatics platform^1^ to identify the overrepresented biological processes and metabolic pathways.

Fungal gene products with potential access to host substrates were identified in two steps. First, the 453 differentially expressed genes were screened for RefSeq product names containing protease, peptidase, lipase, phospholipase, esterase, hydrolase, glycosidase, chitinase, nuclease, amidase, or sulfatase, using the *M. restricta* KCTC 27527 annotation (ASM329048v1). Second, the protein sequences of the 16 retained genes were analyzed with SignalP 6.0 in Eukarya mode. A protein was classified as secreted when the Sec/SPI probability exceeded 0.5. InterPro domain assignments were taken from the same genome annotation. No gene product was tested functionally, so all assignments are predictions.

### 2.11 Statistical analysis

All statistical analyses were performed using R statistical software (version 4.0.5, R Foundation for Statistical Computing, Vienna, Austria). Thickness, cytokine, viability, and trypsin data were analyzed by two-way analysis of variance with *S. aureus* and *M. restricta* as factors. Pairwise comparisons were made with Holm correction, and groups sharing a letter in the figures do not differ at *p* < 0.05. Layer-resolved filaggrin intensity was analyzed by two-way ANOVA with experimental condition and epidermal layer as factors, followed by Tukey’s *post-hoc* test. The SC fraction of total filaggrin was analyzed by one-way ANOVA followed by Tukey’s *post-hoc* test. Blinded histological scores were summarized descriptively. Exploratory Spearman correlations were calculated across the four group means without inferential testing. Spearman coefficients calculated from the four group means described the direction of associations and were not tested for significance. Data are presented as mean ± SD unless otherwise stated. Sample sizes and replicate structures are specified in the corresponding Methods subsections and figure legends. Fungal RNA-seq analysis included two independent biological replicates for each of the MR and MIX groups. Differential gene expression between MIX and MR was analyzed using edgeR with a negative binomial generalized linear model. Genes with an absolute fold change ≥ 2 and an unadjusted *p* < 0.05 were classified as differentially expressed.

## 3 Results

### 3.1 Spatial architecture of microbial-induced epidermal reorganization

**FIGURE 1:**
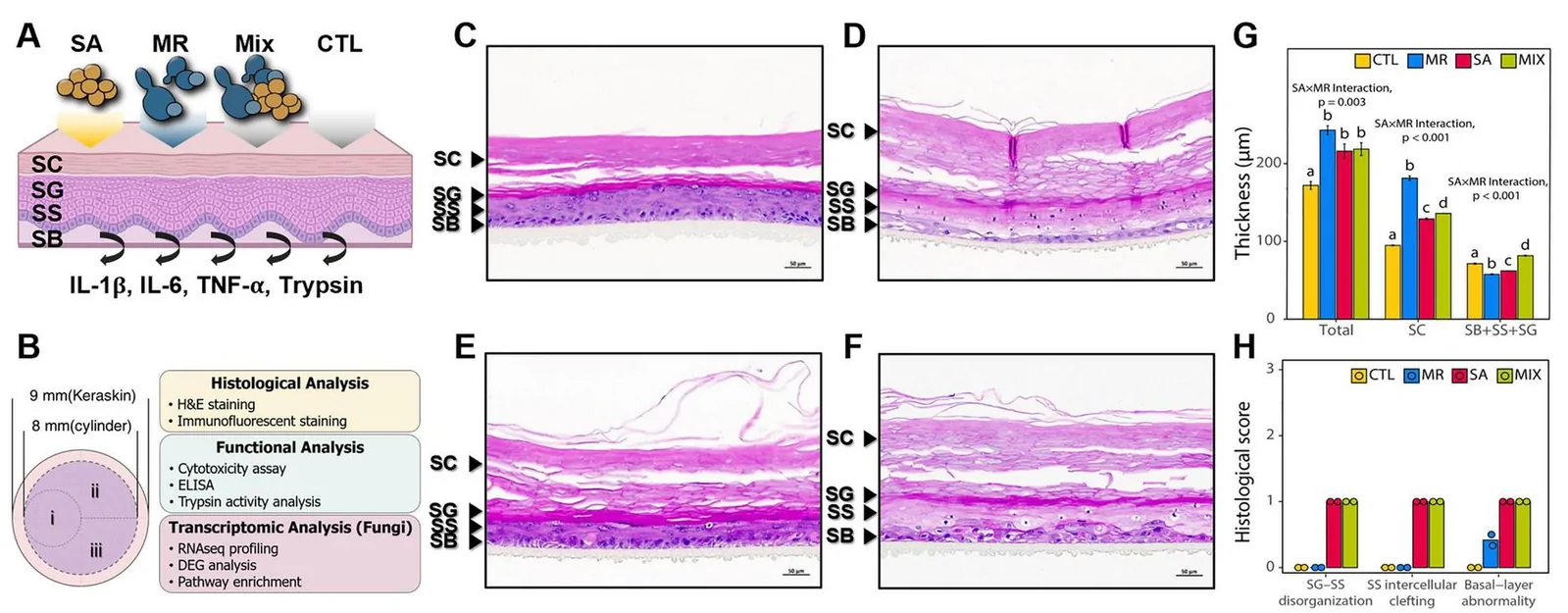
Experimental design and spatial remodeling of 3D human epidermis after microbial colonization. **(A)** Schematic of 48-h colonization with *Staphylococcus aureus* (SA), *Malassezia restricta* (MR), or both (MIX); untreated controls (CTL). Spatially confined inoculation and post-harvest multi-parameter sampling are shown. **(B)** An 8-mm biopsy captured the colonized zone and was subdivided for histological, functional, and transcriptomic assays. Transcriptomic analysis used *M. restricta* reads from the MR and MIX groups only. **(C–F)** Representative H&E images of panel **(C)** CTL, **(D)** MR, **(E)** SA, and **(F)** MIX. Epidermal layers are indicated: stratum corneum (SC), stratum granulosum (SG), stratum spinosum (SS), and stratum basale (SB). Scale bar: 50 μm. **(G)** Quantification of total epidermal, SC, and viable-layer (SG + SS + SB) thickness. Bars show the mean ± SD of ten measurements from two independent RHE inserts per condition. Data were analyzed by two-way ANOVA followed by Holm-adjusted pairwise comparisons. Different letters indicate adjusted *p* < 0.05; SA × MR interaction *p*-values are shown. **(H)** Blinded semi-quantitative scores (0–3) for SG–SS disorganization, SS intercellular clefting, and basal-layer abnormality across 28 coded fields in total, with two RHE inserts per condition. Dots represent the mean score for one RHE insert. Scores were summarized descriptively.

We established a three-dimensional RHE to examine the polymicrobial interactions between *S. aureus* (SA) and *M. restricta* (MR) under mono- and co-colonization (MIX) conditions, compared to untreated controls (CTL) (Figure 1A). After incubation for 48 h, 8-mm biopsies were subdivided for parallel histological, functional (cytotoxicity, cytokines, and trypsin activity), and transcriptomic analyses, enabling correlation between molecular and phenotypic outcomes within identical tissue samples (Figure 1B). The 3D-RHE enabled layer-specific characterization of polymicrobial barrier disruption mechanisms. Specifically, histological analyses revealed fundamentally distinct patterns of architectural remodeling between the single-species and co-colonization conditions (Figures 1C–F). While control tissues (Figure 1C) maintained physiological stratification with defined SC, stratum granulosum (SG), spinosum (SS), and basale (SB) boundaries, microbial colonization induced treatment-specific structural perturbations that were quantifiable through digital morphometry (Figure 1G). Specifically, MR triggered pronounced hyperkeratosis with a 41.6% increase in total epidermal thickness when compared to that in CTL, predominantly through SC expansion (Figures 1D, G). SA induced mild SC perturbation and focal SS cohesion loss; this was accompanied by a modest yet statistically significant increase in the overall epidermal thickness, which remained the least pronounced among all conditions (Figures 1E, G, H). Notably, the MIX condition generated a unique architectural phenotype combining SC hyperkeratosis with increased viable-layer thickness, a pattern absent in either monoculture condition (Figures 1F, G). The viable layer thickness (SG + SS + SB) increased exclusively under co-colonization compared with CTL, despite comparable total thickness to MR. Two-way analysis of variance gave significant SA × MR interactions for total thickness (*p* = 0.003), SC thickness (*p* < 0.001), and viable-layer thickness (*p* < 0.001). Blinded scoring of 28 coded fields showed no SG–SS disorganization and no SS intercellular clefting in CTL or MR (Figure 1H). Both features were present in all scored SA and MIX fields at grade 1. Basal-layer abnormality was absent in CTL, present at grade 1 in three of seven MR fields, and present at grade 1 in all SA and MIX fields.

### 3.2 Layer-specific filaggrin redistribution

**FIGURE 2:**
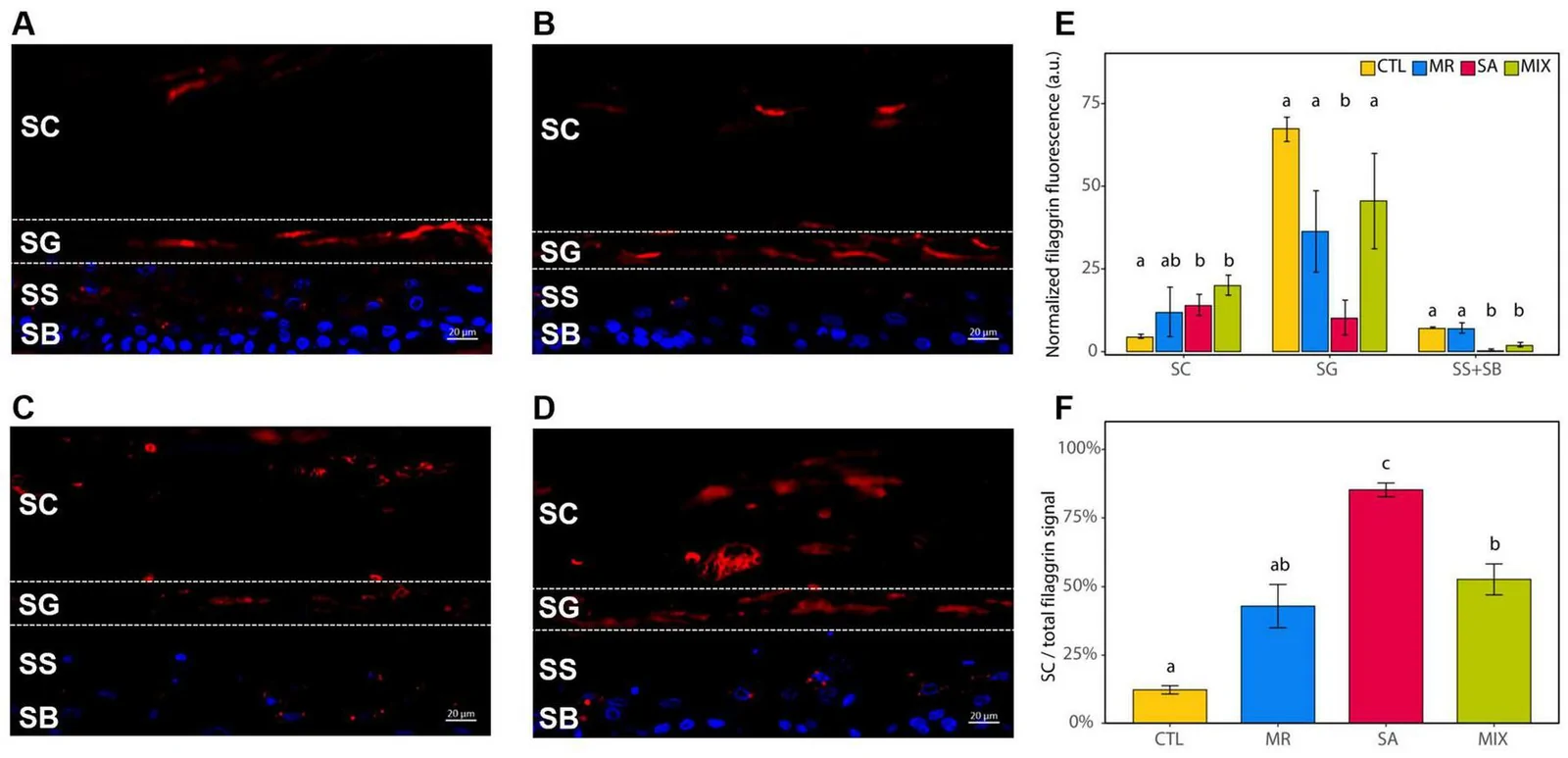
Layer-resolved filaggrin intensity across CTL, MR, SA, and MIX. **(A–D)** Immunofluorescence of filaggrin (red) and nuclei (DAPI, blue) in CTL, MR, SA, and MIX RHE. Scale bar: 20 μm. **(E)** Area-normalized filaggrin fluorescence intensity in the SC, SG, and combined SS + SB. CTL, MR, SA, and MIX were compared separately within each epidermal layer. Quantification used thresholded masks (Supplementary Figure 1) and was normalized to unit area. **(F)** SC fraction of total epidermal filaggrin signal calculated as the integrated fluorescence signal in the SC divided by the summed integrated fluorescence signal in the SC, SG, and SS + SB. Values are means ± SD from five non-overlapping fields per sample. Tukey-adjusted pairwise comparisons of estimated marginal means were performed among CTL, MR, SA, and MIX within each epidermal layer. Groups that do not share a letter differ at adjusted *p* < 0.05.

Filaggrin is an epidermal differentiation protein that aggregates keratin filaments and supports stratum corneum hydration and barrier integrity. Filaggrin intensity was measured separately in the SC, SG, and SS + SB, and the four conditions were compared within each layer (Figure 2, Supplementary Figure 1). CTL tissues exhibited physiological filaggrin localization concentrated in the SG with moderate SC extension, indicating normal profilaggrin processing (Figures 2A, E). MR induced a redistribution signature in the tissues with the same directional shift as SA although no layer differed from CTL (Figures 2B, C, E, Supplementary Figure 2). MIX increased SC signal and reduced SS + SB signal relative to CTL (Tukey-adjusted *p* = 0.0013 and 0.021, respectively), while SG signal did not differ from CTL (Tukey-adjusted *p* = 0.570; Figures 2D, E). As an index of spatial distribution, the SC fraction of the total filaggrin signal was calculated (Figure 2F). This fraction was 12% in CTL, 43% in MR, 85% in SA, and 52% in MIX. SA differed from all other conditions, and MIX differed from CTL but not from MR. Total filaggrin signal integrated across the epidermis did not differ significantly among the four conditions (one-way ANOVA, *p* > 0.05; Supplementary Figure 2).

### 3.3 Cytokine release and cell viability track with *S. aureus*

**FIGURE 3:**
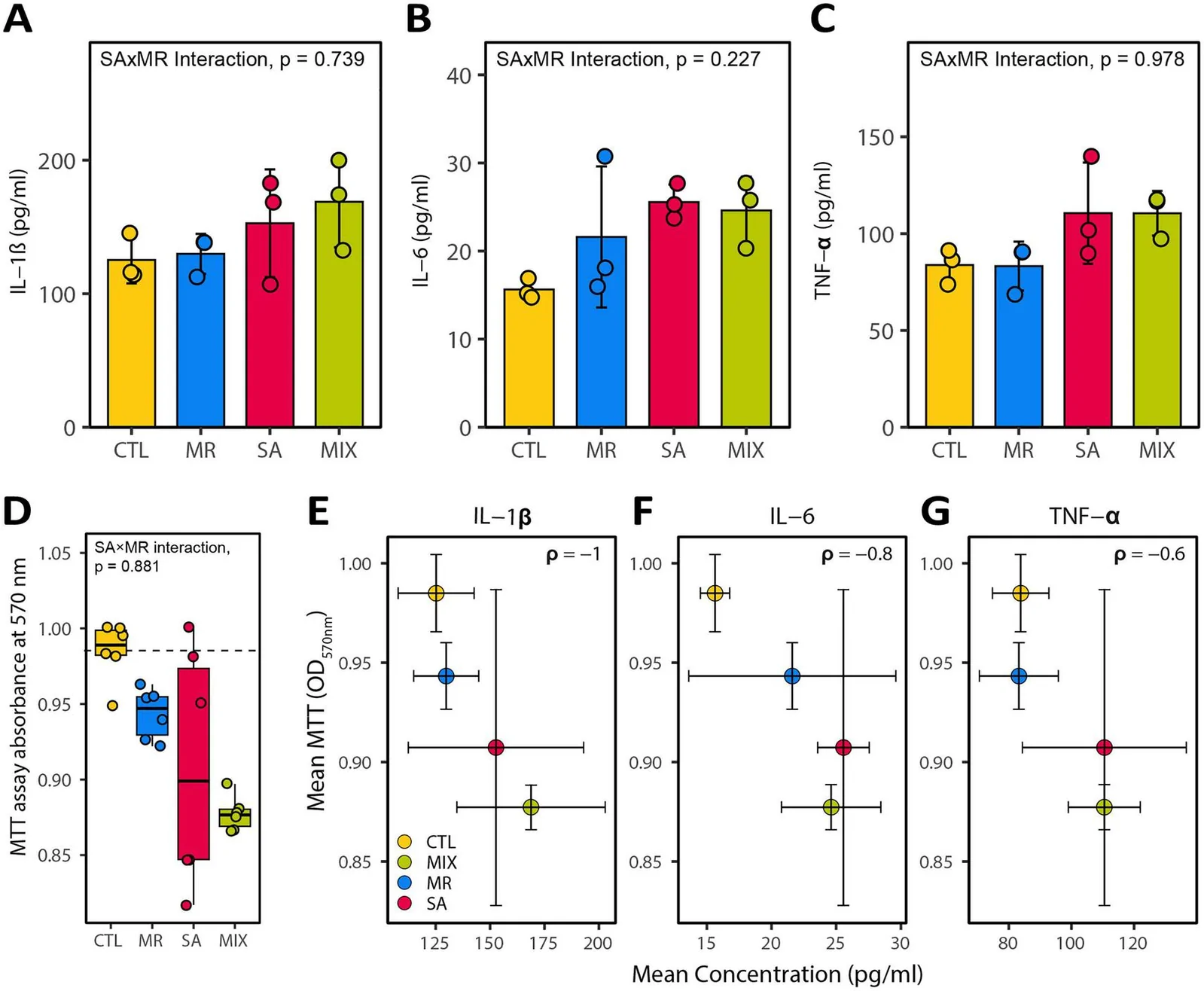
Cytokine concentrations and keratinocyte viability after microbial colonization. **(A–C)** Concentrations of IL-1β, IL-6, and TNF-α in culture supernatants at 48 h (ELISA). Bars represent the mean ± SD, and individual points represent independent RHE tissues. Data were analyzed using two-way ANOVA with SA and MR as fixed factors. The displayed *p*-values correspond to the SA × MR interaction term, which tests whether the response observed in the MIX deviates from additivity. **(D)** Cell viability assessed by MTT assay. Data are shown as box plots (median, IQR, range); the dashed line indicates the CTL mean. The blank control (buffer only) yielded OD = 0.04. The displayed *p*-value represents the SA × MR interaction from two-way factorial ANOVA. **(E–G)** Scatter plots illustrating the relationship between mean cytokine levels (*x*-axis) and mean MTT absorbance (*y*-axis) across treatment groups. Dots represent group means; horizontal error bars indicate the standard deviation (SD) of cytokine levels, and vertical error bars indicate SD of MTT absorbance. Spearman’s correlation coefficients (ρ) are shown in each panel.

Cytokine profiling revealed distinct inflammatory signatures that distinguished the mono- and co-colonization responses (Figures 3A–C). MR selectively induced IL-6 without affecting IL-1β or tumor necrosis factor-α (TNF-α) levels, indicating fungal-specific activation of epithelial inflammatory pathways. SA exhibited broad cytokine upregulation encompassing all three inflammatory mediators, although substantial inter-replicate variability was observed (coefficient of variation: 7.8%–26.4%), which suggested the presence of heterogeneous bacterial colonization patterns within the tissue architecture. MIX values were comparable to SA for all three cytokines. Two-way analysis of variance gave no significant SA × MR interaction for IL-1β (*p* = 0.739), IL-6 (*p* = 0.227), or TNF-α (*p* = 0.978), so the cytokine response was consistent with an additive combination of the two single-species effects.

Metabolic viability assessment via the MTT reduction assay revealed treatment-specific patterns (Figure 3D). CTL maintained baseline metabolic activity (OD_570_ = 0.99 ± 0.02), and MR showed a mean metabolic activity of 0.94 ± 0.02. SA exhibited bimodal toxicity distribution with either minimal impact or depletion, reflecting the heterogeneous colonization observed in the cytokine profiles. MIX showed the lowest mean viability (0.88 ± 0.01). The SA × MR interaction was not significant [two-way ANOVA, *F*(1, 4) = 0.026, *p* = 0.881].

Correlation analysis revealed negative associations between inflammatory mediators and cellular viability across the colonization groups (Figures 3E–G). Spearman’s rank correlations (*n* = 4 groups) demonstrated strong negative trends for IL-1β (ρ = −1.0), IL-6 (ρ = −0.8), and TNF-α (ρ = −0.6), suggesting that increased inflammatory responses are associated with reduced barrier integrity under polymicrobial conditions. The three cytokine-viability relationships were monotonic in the same direction. Each coefficient was calculated from four group means, so the values indicate direction only and were not tested for significance.

### 3.4 Co-colonization reprograms *M. restricta* energy metabolism and stress-response pathways

**FIGURE 4:**
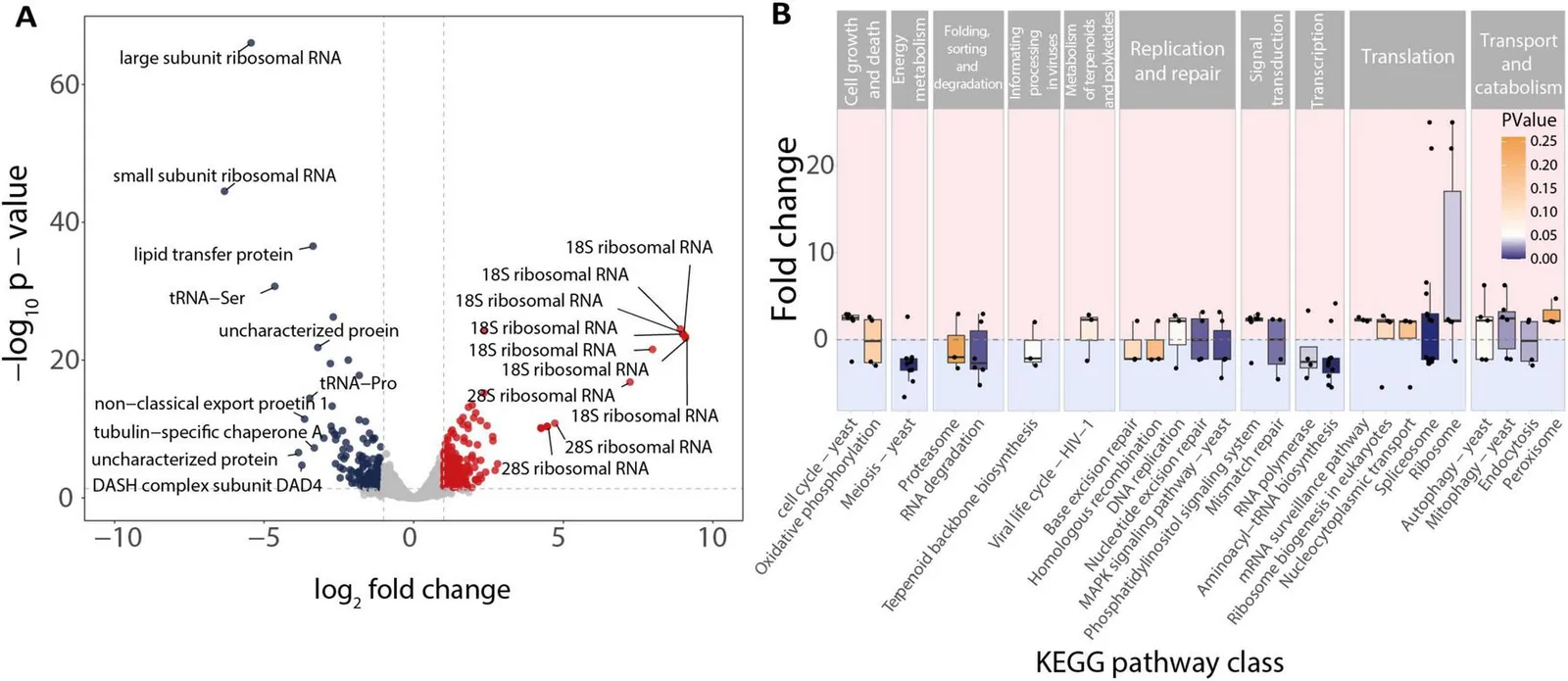
Differential gene expression and KEGG pathway mapping of *M. restricta* under co-colonization with *S. aureus*. **(A)** Volcano plot of DEGs in *M. restricta* under co-colonization, annotated with the top 10 upregulated and downregulated genes ranked by log_2_ fold change. Red and blue points mark up- and downregulated genes at | log_2_FC| ≥ 2 with *p* < 0.05; dashed lines indicate these thresholds. **(B)** KEGG pathway enrichment of DEGs. Each box plot shows the distribution of fold changes for genes mapped to a pathway; color intensity indicates enrichment significance (*p*-value).

Transcriptomic profiling of MR recovered from co-colonized tissues revealed that metabolic restructuring was absent under monoculture conditions. RNA-seq analysis identified 453 differentially expressed genes (DEGs) (256 upregulated and 197 downregulated), showing consistent transcriptional alterations in the metabolic and stress-response pathways (Figure 4A). KEGG (Kyoto Encyclopedia of Genes and Genomes) pathway enrichment analysis revealed the coordinated suppression of oxidative phosphorylation, indicating diminished mitochondrial respiration under interkingdom competition (Figure 4B and Supplementary Table 3). Conversely, ribosomal biogenesis genes showed paradoxical upregulation, with an increase in core ribosomal RNAs (*MRET_r0002* and *MRET_r0004*) and structural proteins (*MRET_1176* and *MRET_0569*), but a decrease in maturation factors (*MRET_2856* and MRET_2527), indicating the selective maintenance of translational capacity despite metabolic constraints (Supplementary Figure 3). This metabolic reprogramming coincides with the activation of stress adaptation pathways. Specifically, autophagy (*MRET_3209*, *MRET_3832*, and *MRET_3308*) and mitophagy components (*MRET_1181* and *MRET_3746*) were upregulated in conjunction with MAPK signaling elements (*MRET_4240*, *MRET_1181*, *MRET_3220*, and *MRET_2445*), whereas RNA degradation and proteasomal pathways were suppressed. This transcriptional signature that reduces energy metabolism, coupled with an enhanced stress response and selective translation, indicates adaptive metabolic dormancy rather than starvation-positioning MR for sustained virulence factor production despite bacterial competition.

### 3.5 Hydrolase-encoding genes among the co-colonization-responsive DEGs

To identify fungal gene products that could act on host substrates, we screened the 453 differentially expressed genes for hydrolase annotations and for predicted secretion. Sixteen genes carried a RefSeq product name indicating protease, peptidase, lipase, or other hydrolase activity (Supplementary Table 4). Only two of these carried a predicted signal peptide, and both decreased under co-colonization. These were a GDSL-like lipase or acylhydrolase (*MRET_3994*, fold change −2.13) and a glycoside hydrolase family 16 protein (*MRET_1767*, fold change −2.57). No gene encoding a predicted secreted protease was differentially expressed. The remaining 14 genes carried no predicted signal peptide, so classical secretion is not predicted for their products. This group included a zinc metalloprotease (*MRET_2360*, fold change 2.13) whose InterPro assignment places it in the M16 peptidase family, together with four deubiquitinases. Both functions act on intracellular substrates and fit the protein turnover indicated by the metabolic shift described above. The transcriptional response of *M. restricta* to *S. aureus* therefore did not include upregulation of a secreted hydrolase.

### 3.6 Trypsin-like activity increases specifically under co-colonization

**FIGURE 5:**
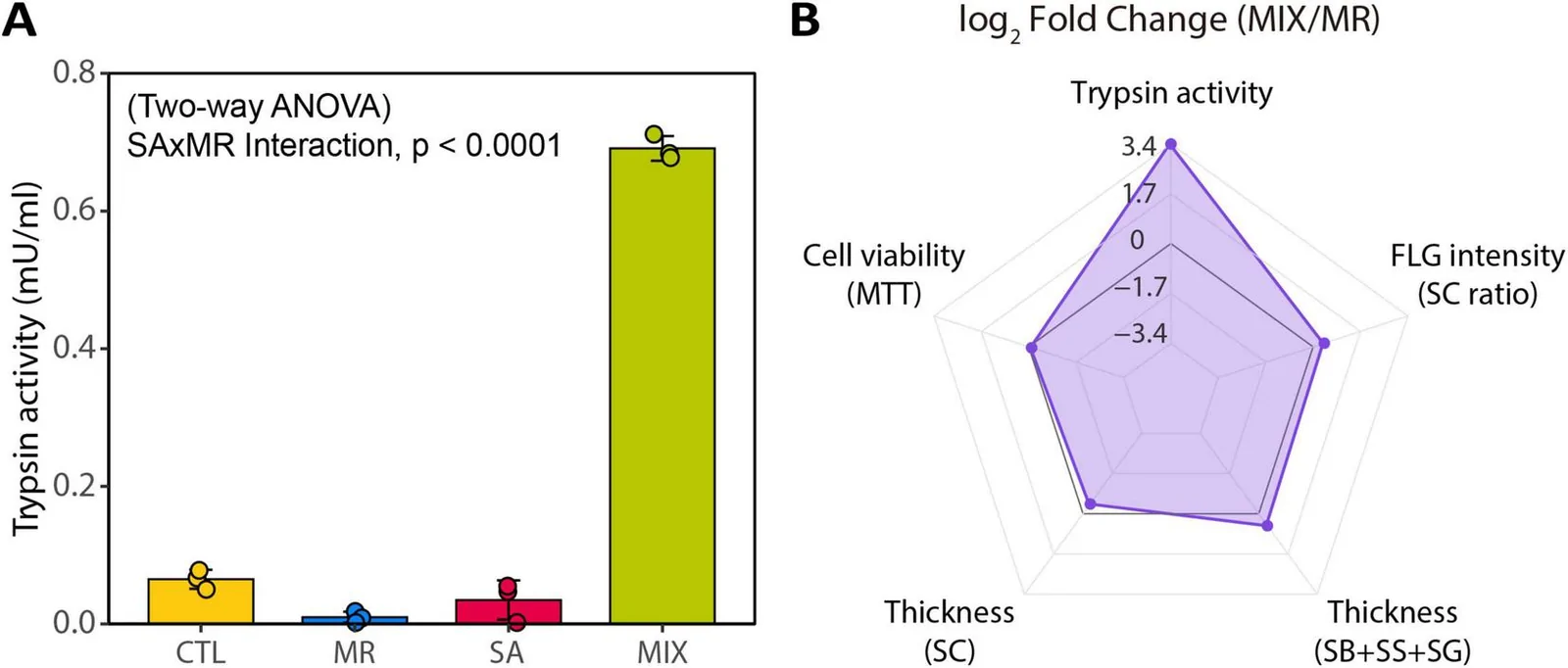
Increased trypsin-like activity during co-colonization and descriptive summary of concurrent molecular and tissue-level changes. **(A)** Trypsin-like activity in CTL, MR, SA, and MIX tissues. Bars indicate the mean ± SD and circles represent independent tissue replicates (*n* = 3). Two-way ANOVA detected a significant SA × MR interaction (*p* < 0.0001). **(B)** The radar plot summarizes barrier-related phenotypes. For filaggrin, the metric is the SC fraction of total epidermal filaggrin fluorescence (SC/total), not absolute intensity; other metrics include stratum corneum thickness, viable epidermal thickness (SG + SS + SB), MTT-based viability, and trypsin activity. The bold ring denotes no change (log_2_FC = 0).

Trypsin-like activity was quantified in tissue lysates from all four conditions (Figure 5A). Mean activity was 0.065 mU/mL in CTL, 0.010 mU/mL in MR, 0.035 mU/mL in SA, and 0.69 mU/mL in MIX. Activity in MIX was 10.6-fold that of CTL and 20-fold that of SA. The SA × MR interaction was significant [two-way ANOVA, *F*(1, 8) = 1093.65, *p* < 0.0001]. Neither MR nor SA raised activity above CTL, so the increase was observed only when both species were present. Under MIX, viable-layer thickness and the SC fraction of the filaggrin signal increased. SC thickness was unchanged and cell viability decreased (Figure 5B).

Trypsin-like activity was correlated with the barrier readouts across the four condition means (Supplementary Figure 4). Activity varied inversely with SC thickness (Spearman rank correlation, ρ = −0.6) and with cell viability (ρ = −0.6), and positively with SC filaggrin intensity (ρ = 0.6) and with the three cytokines (ρ = 0.6–1.0). Each coefficient was calculated from four group means, so the values indicate direction only and were not tested for significance. No differentially expressed gene encoded a predicted secreted protease (Section “3.5 Hydrolase-encoding genes among the co-colonization-responsive DEGs”). The source of the trypsin-like activity was not identified in this study.

## 4 Discussion

This study identified co-colonization with *M. restricta* and *S. aureus* altered epidermal architecture and increased trypsin-like activity in a three-dimensional human epidermal model. Co-colonization increased viable-layer thickness and trypsin-like activity beyond either mono-colonization. Cytokine release and loss of cell viability instead tracked with *S. aureus*, consistent with the absence of significant SA × MR interactions for these endpoints. *M. restricta* alone increased SC thickness, while the filaggrin analyses showed layer-specific redistribution rather than total filaggrin loss (Figures 1G, 2E). Two endpoints departed from an additive combination of the single-species effects. Viable-layer thickness and trypsin-like activity increased only under co-colonization, and each showed a significant *S. aureus* and *M. restricta* interaction. Cytokine release and loss of cell viability showed no interaction and tracked with *S. aureus*. Barrier-related changes in this model therefore separate into an *S. aureus*-driven component and a co-colonization-specific component that neither species reproduced alone. We did not measure toxin distribution or bacterial virulence, so the mechanism linking the two-species condition to the raised proteolytic activity remains undetermined.

Our transcriptomic analysis revealed that *M. restricta* underwent coordinated metabolic reprogramming in response to bacteria. Specifically, genes involved in oxidative phosphorylation, amino acid biosynthesis, and RNA turnover were broadly suppressed, whereas those involved in stress-response pathways such as autophagy, mitophagy, and protein repair were upregulated (Supplementary Figure 3). This shift reflects a low-energy stress-adaptive survival mode that favors persistence in competitive or inflammatory niches (Sukumaran et al., 2022). Genes for internal amino acid production were downregulated while several hydrolase-encoding genes increased (Supplementary Table 4). Among the 16 hydrolase-annotated differentially expressed genes, only two carried a predicted signal peptide, and both decreased. The upregulated hydrolases included a zinc metalloprotease of the M16 family and four deubiquitinases which act on intracellular substrates. This pattern is consistent with increased intracellular protein turnover rather than with increased degradation of host structural proteins.

Elevated activity of host serine proteases, including kallikreins and trypsin-like enzymes, is a consistent feature of inflammatory dermatoses (Nomura et al., 2020). These proteases contribute to barrier disruption, filaggrin cleavage, and inflammation (Moosbrugger-Martinz et al., 2022; Zani et al., 2021). Most work on their activation has examined single organisms. Here, trypsin-like activity rose more than tenfold under co-colonization and stayed at baseline under either species alone. The requirement for both organisms is itself informative because it places the trigger in the two-species condition rather than in a product of either microbe. Our screen of the fungal transcriptome narrows the candidate space further, since no differentially expressed gene encoded a predicted secreted protease. This leaves host-derived and bacterial proteases as the main candidates. The major secreted proteases of *S. aureus* are a glutamyl endopeptidase, a metalloprotease, and cysteine proteases, none of which have trypsin-like specificity (Rasquel-Oliveira et al., 2025). Epidermal kallikreins such as KLK5 and KLK14 are trypsin-like and are secreted as zymogens requiring proteolytic activation (Bekassy et al., 2022; Zani et al., 2021), which makes them plausible contributors. Inhibitor and knockdown experiments could separate these possibilities.

Despite these mechanistic insights, our study has some limitations that warrant further investigation. First, the use of a simultaneous co-colonization model may not fully reflect the temporal dynamics of natural skin colonization, wherein the presence of fungi may precede bacterial overgrowth. Second, our *in vitro* system lacked immune cell components that induce inflammation and tissue remodeling. We measured IL-1β as an inflammasome-associated readout and did not measure IL-1α or IL-1Ra, which limits characterization of the broader epidermal IL-1 response. Although keratinocytes mediate some innate immune responses, they cannot replicate the full spectrum of cutaneous immune signaling. Third, this study focused on only two microbial species, whereas the native skin microbiome comprises a complex community that can modulate or offset these interactions. Consequently, future studies should incorporate immune-competent models, sequential colonization designs, and multispecies microbial communities to better reflect the physiological conditions. These results reframe barrier disruption as a co-colonization-specific property of interkingdom interaction rather than the action of a single species.

**FIGURE 6:**
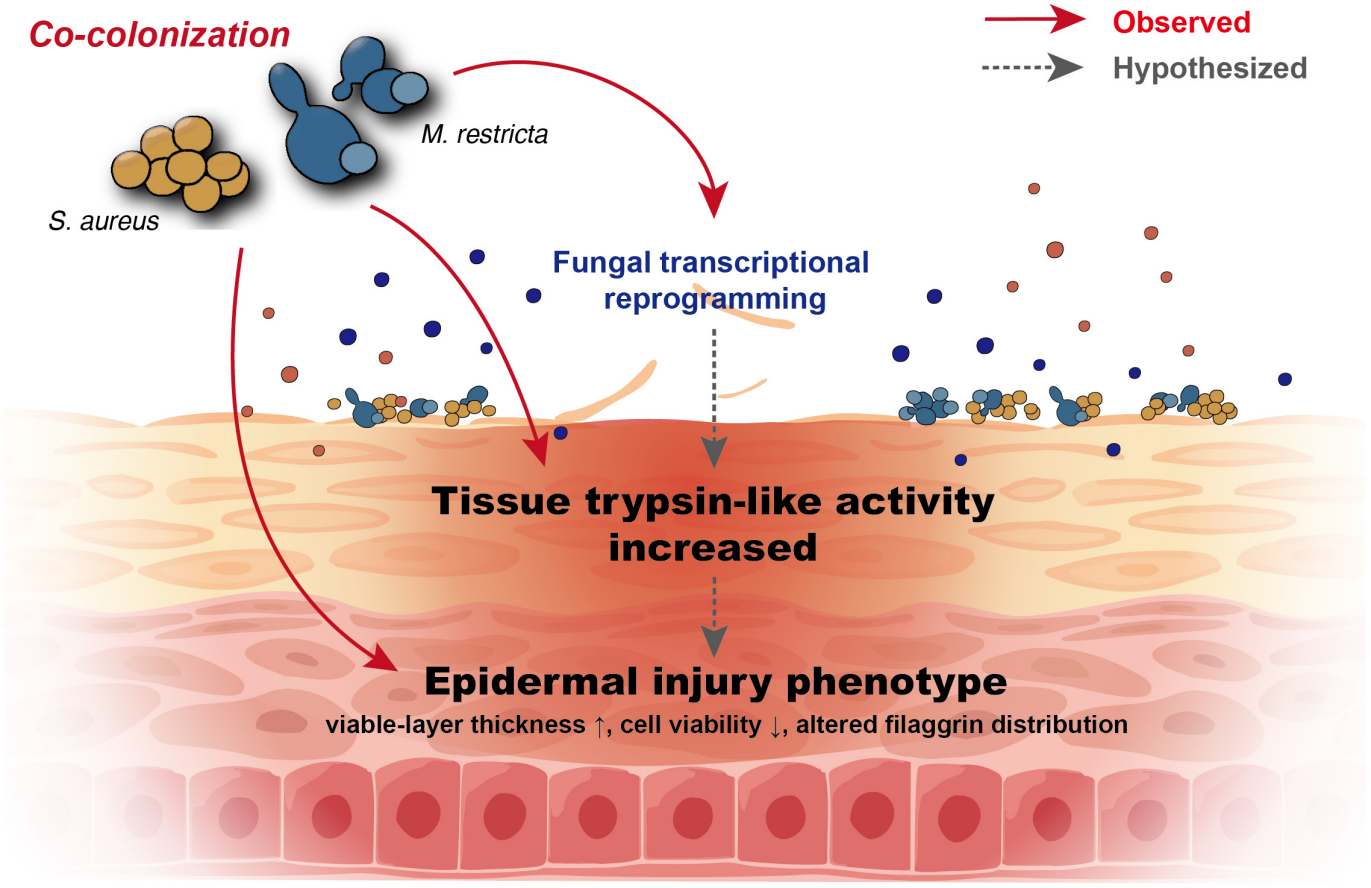
Proposed model of co-colonization-associated epidermal disruption. Co-colonization with *M. restricta* and *S. aureus* was associated with transcriptional reprogramming in *M. restricta*, increased trypsin-like activity in RHE tissues, and changes in epidermal structure and filaggrin distribution. Relative to CTL, MIX tissues showed increased viable-layer thickness and a higher proportion of filaggrin signal in the SC. Layer-resolved analysis further showed increased SC signal and reduced SS + SB signal, whereas SG signal did not differ significantly from CTL. Solid red arrows indicate effects of co-colonization measured in this study. Dashed gray arrows indicate hypothesized relationships that were not tested.

In summary, co-colonization by *M. restricta* and *S. aureus* produced two outcomes that neither species produced alone (Figure 6). Trypsin-like activity rose more than tenfold only when both were present, and viable-layer thickness increased only under co-colonization. Cytokine release, loss of cell viability, and filaggrin redistribution tracked with *S. aureus*. This concept provides a new perspective on microbial pathogenicity as an emergent property of interkingdom interaction which may help explain why polymicrobial colonization tracks with severity in inflammatory skin disease.

## Data Availability

The original contributions presented in this study are included in the article/Supplementary material, further inquiries can be directed to the corresponding author.

## Footnotes

1
https://davidbioinformatics.nih.gov/
