# Supplementary material for: Bacterial and fungal co-colonization alters Malassezia restricta metabolism and host proteolytic activity in reconstructed human epidermis
Source: Front Microbiol. 2026 Aug 24;17:1927213. doi: 10.3389/fmicb.2026.1927213 (PMC13547511; doi:10.3389/fmicb.2026.1927213)
Supplement: Supplementary file 1 [file Data_Sheet_1.docx]

Supplementary Material

***Staphylococcus aureus* reprograms *Malassezia restricta* metabolism to amplify host proteolysis in reconstructed human epidermis**

**Eun Sun Lyou^1^, Sang-Hyeon Yoo^2^, Ki-Jong Rhee^2^, David R. Johnson^3,4^ and Tae Kwon Lee^1*^**

^1^Department of Environmental & Energy Engineering, Yonsei University, Wonju 26493, Republic of Korea

^2^Department of Biomedical Laboratory Science, Yonsei University, Wonju 26493, Republic of Korea

^3^Department of Environmental Microbiology, Swiss Federal Institute of Aquatic Science and Technology (Eawag), Dübendorf 8600, Switzerland

^4^Institute of Ecology and Evolution, University of Bern, Bern 3012, Switzerland

***Correspondence:** Tae Kwon Lee, Department of Environmental & Energy Engineering, Yonsei University, Wonju, 26493, Republic of Korea, E-mail: [tklee@yonsei.ac.kr](mailto:tklee@yonsei.ac.kr), Phone: +82-33-760-2446, Fax: +822-33-760-5286

**Supplementary Figure 1. Threshold-based binarization for layer-resolved filaggrin mapping.** Representative binary masks from CTL, MR, SA, and MIX RHE. Filaggrin-positive pixels are shown in black. Masks were generated by intensity thresholding of the filaggrin channel and used to quantify signal abundance and distribution across epidermal layers.

**Supplementary Figure 2. Section-integrated filaggrin fluorescence across the epidermis (CTL, MR, SA, and MIX).** Total filaggrin fluorescence was integrated across the full epidermal thickness for each group (CTL, MR, SA, MIX). Values are means ± SD from five fields per sample. One-way ANOVA with Tukey’s post hoc test; different letters indicate significant differences (p<0.05).

**Supplementary Figure 3. Heatmap of KEGG-mapped genes grouped by functional categories (amino acid/protein metabolism, energy metabolism, stress/adaptation).** Pathway labels in bold with asterisks denote significantly enriched pathways (*p*<0.05). Each tile represents one gene; color indicates log₂ fold change, and asterisks within tiles mark gene-level significance (**p*<0.05, ***p*<0.01, ****p*<0.001).

**Supplementary Figure 4. Pairwise Spearman correlation matrix of trypsin activity, barrier-related phenotypes, and inflammatory cytokines.** Correlations were computed using condition-level means (one mean within CTL, MR, SA, and MIX). Variables include trypsin activity; epidermal thickness of the stratum corneum (SC) and the viable layer (SG+SS+SB); the filaggrin SC fraction of total epidermal fluorescence (SC/total); MTT-based cell viability; and IL-1β, IL-6, and TNF-α levels. The lower triangle displays Spearman’s ρ as color-coded ellipses; cell annotations show nominal *p*-values. Correlations are provided for descriptive interpretation only due to limited replication at the group-mean level.
